# Editorial: Neurobehavioral toxicity induced by environmental contaminants: mechanisms of neurodegeneration and neuroprotection, Volume II

**DOI:** 10.3389/fphar.2023.1255471

**Published:** 2023-07-28

**Authors:** Daniel J. Dorta, Danielle P. Oliveira, Alline C. Campos, Michael Aschner

**Affiliations:** ^1^ Department of Chemistry, Faculty of Philosophy, Sciences and Letters of Ribeirão Preto, University of São Paulo, São Paulo, Brazil; ^2^ Department of Clinical Analyses, Toxicology and Food Science, School of Pharmaceutical Sciences of Ribeirão Preto, University of São Paulo, São Paulo, Brazil; ^3^ Department of Pharmacology, School of Medicine of Ribeirão Preto, University of São Paulo, São Paulo, Brazil; ^4^ Department of Molecular Pharmacology, Albert Einstein College of Medicine, New York City, NY, United States

**Keywords:** enviromental contaminats, heavy metals, neurotoxicity, reactive oxygen species (ROS), neurodegenaration

Environmental contaminants pose a significant threat to human health, particularly in the context of neurotoxicity. Heavy metals, herbicides, and persistent organic pollutants have been extensively studied and associated with neurodegeneration and cognitive impairments. The goal of this series of manuscripts, which was published in the scientific journal Frontiers in Toxicology, is to provide a comprehensive understanding on the mechanisms underlying neurotoxicity induced by environmental pollutants. These articles shed light on the complicated processes by which pollutants exert their harmful effects, by investigating the intricate interplay of multiple pathways such as oxidative stress, mitochondrial dysfunction, inflammation, and epigenetic changes.

One of the fundamental mechanisms by which environmental contaminants induce neurotoxicity is through the generation of oxidative stress. Contaminants such as lead, mercury, and cadmium induce reactive oxygen species (ROS), which in turn can trigger lipid peroxidation, DNA damage, and protein oxidation within neural cells. This oxidative stress not only contributes to neuroinflammation, but also activates apoptotic pathways, ultimately leading to neuronal cell death. Further exploration of the specific mechanisms by which contaminants induce oxidative stress and the resulting consequence on neurological function provides valuable insights into putative therapeutic interventions.

Another crucial mechanism underlying neurotoxicity is mitochondrial dysfunction. Many contaminants disrupt mitochondrial function by altering mitochondrial membrane potential, impairing ATP production, and promote mitochondrial ROS generation. Therefore, cellular energy deficits occur, leading to chronic inflammation and neuronal damage. The exploration of how various contaminants interact with mitochondria and the subsequent implication for neuronal health offers insight into the multifaceted nature of neurotoxicity.

Inflammation serves as a significant pathway by which environmental contaminants exert their neurotoxic effects. Pesticides and persistent organic pollutants are known to activate the immune system, resulting in the release of proinflammatory cytokines and chemokines. These inflammatory mediators can disrupt normal neuronal signaling, giving rise to cognitive deficits and neurodegeneration. Unraveling the intricate mechanisms by which contaminants induce inflammation and the subsequent impact on neural function provides crucial insights into the development of targeted therapies.

Emerging evidence suggests that epigenetic modifications play a pivotal role in mediating neurotoxicity induced by environmental contaminants. Contaminants such as bisphenol A and phthalates have been found to alter DNA methylation, histone modifications, and microRNA expression, leading to changes in gene expression that can affect neuronal development and function. Understanding the epigenetic mechanisms involved in neurotoxicity provides novel avenues for interventions that might mitigate the adverse effects of contaminants on the nervous system.

**FIGURE 1:**
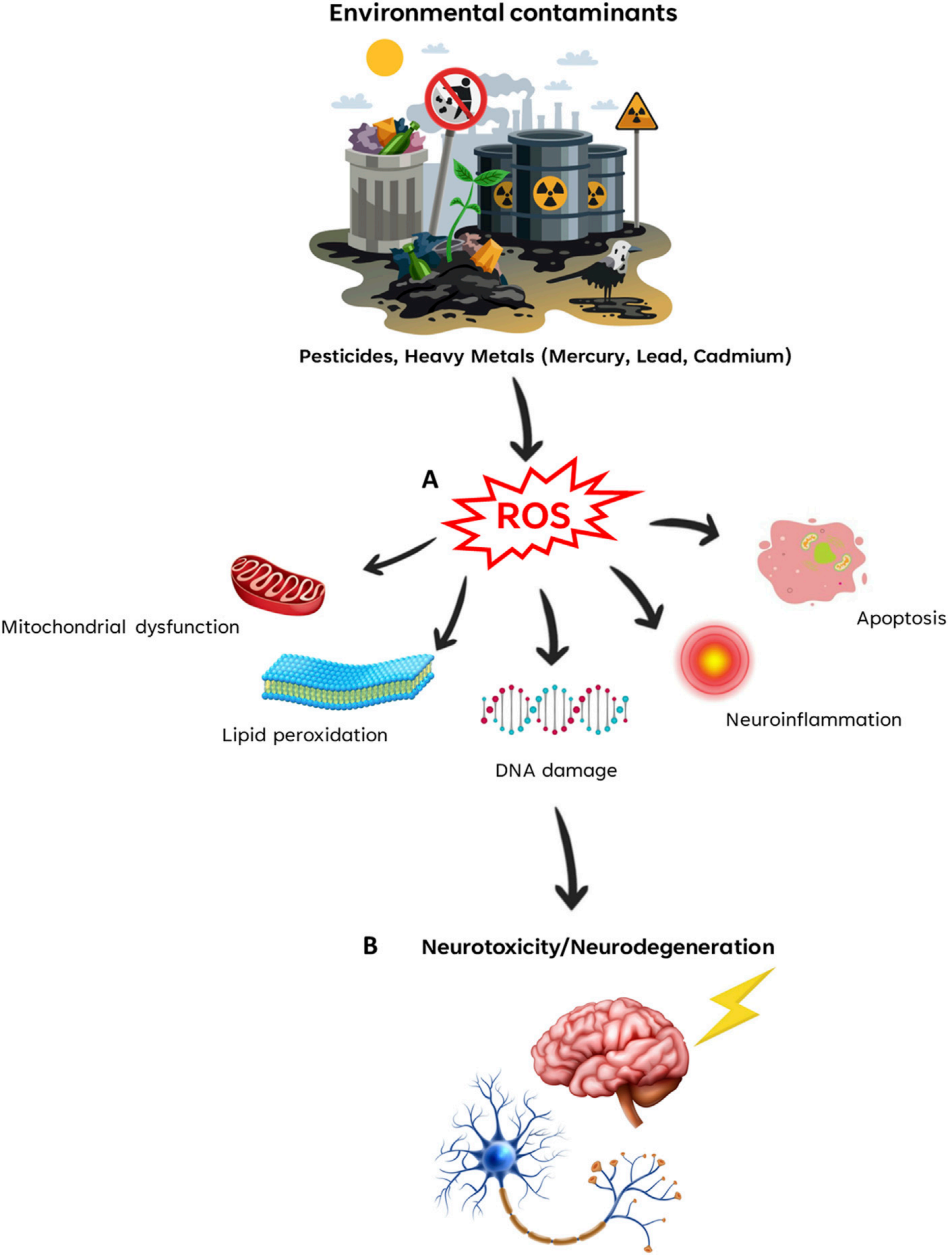
Environmental contaminants-induced neurotoxicity. Environmental contaminants induce the production of ROS (Reactive Oxygen Species) **(A)**, which triggers several cellular mechanisms that leads to neurotoxicity and neurodegeneration **(B)**.

By focusing on oxidative stress, mitochondrial dysfunction, inflammation, and epigenetic modifications, the article Research Topic aims to provide a comprehensive understanding of the complex interplay between contaminants and the nervous system (Figure 1). This knowledge not only expands our understanding of the adverse effects of environmental contaminants on neurobehavioral health, but also opens new avenues for the development of targeted therapeutic strategies.

The present volume is designed to address contemporary issues in neurodevelopmental neurotoxicity and is comprised of 4 papers which address diverse topics. These include an original research article “Isoimperatorin therapeutic effect against aluminum induced neurotoxicity in albino mice”. The study found that IMP might be a promising treatment option for neurotoxicity and neurodegenerative diseases, such as Alzheimer’s disease and Parkinson’s disease, which are associated with neuro-inflammation and oxidative stress (Rajendran et al.).

A second article intitled: “Neuroprotection of Gastrodia elata polyphenols against H2O2-induced PC12 cell cytotoxicity by reducing oxidative stress” is an original research article that focuses on the potential of Gastrodia elata polyphenols (GPP) for the prevention and treatment of Alzheimer’s disease. The study found that GPP pretreatment had a protective effect by increasing cell viability, reducing lactate dehydrogenase infiltration, decreasing MDA and increasing intracellular antioxidant enzymes, diminishing reactive oxygen species production and decreasing mitochondrial membrane potential, reducing cell inflammation and decreasing apoptosis (Tan et al.).

Another original research published in this volume “Nicotine alleviates MPTP-induced nigrostriatal damage through modulation of JNK and ERK signaling pathways in the mice model of Parkinson’s disease” found that nicotine reduced MPTP-induced behavioral deficits, dopaminergic neuron loss, oxidative stress, inflammation, and apoptosis in the mice. The authors also found that nicotine modulated the activation of JNK and ERK signaling pathways, which are involved in neuronal survival and death, concluding that nicotine may have neuroprotective effects against MPTP-induced nigrostriatal damage by regulating JNK and ERK signaling pathways (Ruan et al.).

Finally, the series includes a review on “Mechanisms of manganese-induced neurotoxicity and the pursuit of neurotherapeutic strategies”. The authors summarize manganese’s toxicodynamic as well as potential molecular targets for treatment and possible neurotherapeutic agents in the search for treatment (Pajarillo et al.).

As Research Topic Editors of this volume, we hope that the articles published here will make a significant contribution to the advancement of research in the field of neurotoxicology.

